# Language Assessment for Immigration: A Review of Validation Research Over the Last Two Decades

**DOI:** 10.3389/fpsyg.2021.773132

**Published:** 2021-11-11

**Authors:** Don Yao, Matthew P. Wallace

**Affiliations:** Department of English, University of Macau, Macau, China

**Keywords:** language assessment, immigration, validation, review, Assessment Use Argument framework

## Abstract

It is not uncommon for immigration-seekers to be actively involved in taking various language tests for immigration purposes. Given the large-scale and high-stakes nature those language tests possess, the validity issues (e.g., appropriate score-based interpretations and decisions) associated with them are of great importance as test scores may play a gate-keeping role in immigration. Though interest in investigating the validity of language tests for immigration purposes is becoming prevalent, there has to be a systematic review of the research foci and results of this body of research. To address this need, the current paper critically reviewed 11 validation studies on language assessment for immigration over the last two decades to identify what has been focused on and what has been overlooked in the empirical research and to discuss current research interests and future research trends. Assessment Use Argument (AUA) framework of Bachman and Palmer (2010), comprising four inferences (i.e., assessment records, interpretations, decisions, and consequences), was adopted to collect and examine evidence of test validity. Results showed the *consequences* inference received the most investigations focusing on immigration-seekers’ and policymakers’ perceptions on test consequences, while the *decisions* inference was the least probed stressing immigration-seekers’ attitude towards the impartiality of decision-making. It is recommended that further studies could explore more kinds of stakeholders (e.g., test developers) in terms of their perceptions on the test and investigate more about the fairness of decision-making based on test scores. Additionally, the current AUA framework includes only positive and negative consequences that an assessment may engender but does not take compounded consequences into account. It is suggested that further research could enrich the framework. The paper sheds some light on the field of language assessment for immigration and brings about theoretical, practical, and political implications for different kinds of stakeholders (e.g., researchers, test developers, and policymakers).

## Introduction

Interest in language assessment for immigration started approximately two decades ago (Kunnan, 2012a). The ALTE (Association of Language Testers in Europe, 2007) reported that nearly all countries took language proficiency (often represented by the score or level of a test) as a requirement for immigration or citizenship, and it was specifically determined by the cut scores prescribed by immigration authorities. Hence, language proficiency has gradually become a salient issue in the discussion of immigration policy because it is regarded as a pre-entry requirement (Magos and Politi, 2008; Kostakopoulo, 2010; Goodman, 2011; Frost and McNamara, 2018); and immigration-seekers’ application results, even partially based on language proficiency, may still bring about tremendous consequences to both stakeholders and society (Shohamy and Kanza, 2009;  McNamara and Ryan, 2011; Saville, 2012).

Given the high-stakes and large-scale nature of language tests for immigration purposes and the gatekeeping role the cut-off score plays, the validity issues of language tests are of great importance in that the test score is directly linked to immigration-seekers’ application results or visa-grant decisions (Merrifield, 2012). Validity, as a core quality of test appraisal, is given great importance in the arena of language assessment. Typical definitions of validity are interpreted as the correlation of scores on a test with “some other objective measure of that which the test is used to measure” (Bingham, 1937, p. 214); or “in a very general sense, a test is valid for anything with which it correlates” (Guilford, 1946, p. 429). These definitions indicate that validity is a quality mainly related to the appropriate interpretations based on test scores for making certain decisions (Chapelle, 1999; Popham, 2017). Validation is a broader term and refers to the evaluation process to argue for the validity of score-based interpretations, decisions, and consequences (Bachman, 2005; Im et al., 2019; Giraldo, 2020). It plays an indispensable role in the arena of language assessment as it gathers both theoretical and empirical evidence to reveal how an assessment is used as expected and accounts for beneficial uses (Kane, 2006; Chapelle and Voss, 2013). Therefore, it is deemed that validation research usually investigates the validity issues of language tests.

Regarding language assessment for immigration, there is no review paper that has ever summarized the foci and results in terms of validation research. Nevertheless, due to the emergence of validation research on language assessment for immigration purposes, a review paper is warranted to examine what has been focused on and what has been overlooked in previous studies, to discuss current research interests and future research trends, and to reveal potential implications for different groups of stakeholders (e.g., researchers, test developers, and policymakers). From here, the present paper critically reviewed the validation research on language assessment for immigration purposes over the last 20years, and the framework that guides the paper has been first demonstrated.

## The AUA Framework

The validity of an assessment is usually appraised through a validation framework that guides critical discussions or analyses (Pochon-Berger and Lenz, 2014). The Assessment Use Argument (AUA) framework proposed by Bachman and Palmer (2010), carrying both conceptual and practical functions in test evaluation, contains four specific inferences to be made about test use: *assessment records* (the score from an assessment), *interpretations* of the test-taker ability based on their score, *decisions* that are made as a result of the interpretations, and *consequences* of those decisions for stakeholders. The AUA draws inspiration from argumentation structure of Toulmin (2003), whereby general claims describing an ideal use of a test are given under each dimension. When the supportive evidence is provided for a claim (called backing) is greater than the contradictory evidence (called rebuttal), then a test’s use is considered valid. The framework has been briefly introduced in the following sub-sections, and the general claims for each dimension of the framework are situated within the immigration test context.

### Assessment Records

The general claim is that scores or verbal descriptions given for test performance should be consistent across different characteristics of an assessment (e.g., assessment tasks, administrative and scoring procedures, and different groups of test-takers). To provide evidence in support of the *assessment records* inference, researchers inspect the internal consistency reliability estimates among the items on a test and the correlations between test-takers’ performance and the characteristics of the assessment. The general claim is supported when consistency is high. For assessments that require production (e.g., writing tasks), the consistency with multiple raters scored the same performance, or inter-rater reliability, is examined. When there is high agreement among the raters, the general claim is supported. This inference may not be directly linked to language assessment for immigration purposes, but it provides a solid foundation for further validation steps because a strong correlation of test scores could strengthen and consolidate the reliability of the test.

### Interpretations

The general claim is that the interpretations about the ability assessed on a test should be meaningful, impartial, generalizable, relevant, and sufficient. Interpretations are meaningful if an assessment evaluates what it purports to, which is also referred to as validity, is in relation to the construct of an assessment. *Meaningfulness* is largely based on an analysis of the language abilities needed to perform tasks in the target language use (TLU) domain, or the context in which the language used be used. For instance, an immigration test may evaluate the communication ability of a test-taker to successfully complete an in-store purchase through international tasks. Interpretations of test scores are meaningful when there is evidence that the test measures what it purports to measure (also known as validity), and minimizes construct irrelevance and construct under-representation (Messick, 1989, 1996). For example, an immigration test is probably more reasonable to focus on test-takers’ capacities to survive in the country, rather than their academic language abilities.

*Impartiality* pertains to the fairness of the interpretations of an assessment. Interpretations are fair if the test administration (1) gives sufficient access to a test and its preparation materials for all test-takers; (2) utilizes similar procedures across administrations; and (3) avoids biasing in favor of or against a group of test-takers (Kunnan, 2004; Xi, 2010; Wallace, 2018). That said, for immigration authorities, it is essential to provide enough materials on the official website so that immigration-seekers may have the same opportunity-to-learn; in terms of test developers, they need to design items without biasing caused by different personal backgrounds, such as age, gender, religion, and ethnicity (Kunnan, 2004, 2012b).

Score interpretations can be *generalizable* when there is a high degree of correspondence between the assessment tasks and tasks in the TLU domain. Test developers should strive hard for developing tasks in the immigration test that are associated with real-life situations that the immigration-seekers are likely to meet after immigration. Besides, the score interpretations of an assessment should be *relevant* to the decisions to be made. Additionally, the score interpretations ought to provide *sufficient* information for the decisions to be made. To be more specific, immigration authorities should be cautious to set the cut-off scores because they ought to have strong justifications to argue the reason that they set this cut-off score; and test agencies are supposed to provide comprehensive score reports with immigration authorities to make final decisions.

### Decisions

The general claims are that the *decisions* made based on the interpretations should: take into consideration existing community values (i.e., educational and societal values) and legal requirements (i.e., relevant laws, rules and regulations); and be equitable for those stakeholders who are affected. In other words, the decisions should uphold the values of the community making such decisions and be within the confines of the law; additionally, when the same criteria for making decisions are applied to all stakeholders (i.e., test-takers), then the decisions can be considered fair. In contrast, decisions may be unfair if they are made inconsistently or when they favor one group of stakeholders over another. The decisions in the immigration test context are often immigration-seekers’ application results. If cut-off scores of immigration tests are different according to country of origin, the decisions made by the governing body may be considered unfair.

### Consequences

The general claim is that the *consequences* of an assessment should be beneficial to all stakeholders. The beneficence refers to the extent to which the consequences of test use and decisions promote good and are not detrimental to stakeholders (Kunnan, 2004). This claim is somewhat controversial because an assessment may not always elicit positive consequences. Sometimes negative consequences may occur (e.g., the failure of immigration) and the assessment may be deemed detrimental to the stakeholders (Messick, 1989; Shepard, 1997; Stobart, 2001). Therefore, the potential rebuttal is that an assessment will have detrimental consequences for the stakeholders who are affected. To situate the *consequences* inference into the immigration test context, the immediate stakeholders include immigration-seekers or immigrants, government officers or policymakers, test developers, or even instructors at educational agencies. The consequences of decisions of these test scores and their interpretations can mean being allowed to immigrate to a country or being prevented from immigration.

## Selection of Studies

To collect papers, the method of preferred reporting items for systematic reviews and meta-analyses (PRISMA) was adopted (Moher et al., 2009; Shintani and Wallace, 2014; Fan and Yan, 2020). The literature search was intended to identify published studies investigating the validity issues of language tests for immigration purposes. The analysis was carried out by examining the electronic academic database *Google Scholar*. The keywords “language assessment,” “immigration,” and “validity/validation” were input into the database. The time “since 2000” was chosen because the research on language assessment for immigration started at the beginning of a new era, and papers were sorted by relevance.

The first search revealed that 18 published papers were initially retrieved. These studies were further screened through the following dimensions: (1) studies mentioning any validity issues of language tests for immigration purposes; and (2) studies that were research articles but not commentaries or field introductory articles. Of these papers, seven of which did not satisfy the dimensions, and they were, therefore, excluded. The abstracts and full texts of the remaining 11 papers were assessed and all of them were ultimately included because of the relevance to this review paper.

## Existing Studies

There are 11 empirical validation studies on language assessment for immigration purposes. About half of them have been conducted on large-scale language proficiency tests (IELTS and TOEFL) and the other half by locally produced tests [Deutsch-Test für Zuwanderer (DTZ) in Germany, Toets Gesproken Nederlands (TGN) in Holland, and the Canadian English Language Proficiency Index Program (CELPIP) in Canada]. The foci of these validation studies vary broadly, but they consistently produce inferences for one to two inferences of the AUA. The studies are presented in Table 1. What follows is the review of these studies and the findings of which will be interpreted in the context of the AUA dimensions.

**Table 1:** Empirical validation research on language assessment for immigration.

Author(s) and year of study	Test(s)	Focus/Foci of research	AUA inference(s)
*Large-scale language proficiency tests*			
Merrylees, 2003	IELTS	Test-takers’ perceptions	*Consequences*
Ahern, 2009	IELTS	Students’ perceptions	*Consequences*
Merrifield, 2012	IELTS	Immigration authorities’ perceptions	*Consequences*
Rumsey et al., 2016	IELTS	Immigrants’ perceptions	*Consequences*
Frost, 2017	IELTS	Immigrants’ perceptions	*Consequences*
Hoang, 2019	IELTS and TOEFL	Immigration-seekers’ perceptions	*Decisions* and *Consequences*
*Locally produced tests*			
De Jong et al., 2009	TGN	Score consistency, access, bias, and content validity	*Assessment records* and *Interpretations*
Perlmann-Balme, 2011	DTZ	Score consistency, construct validity, and content validity	*Assessment records* and *Interpretations*
Plassmann, 2011	DTZ	Score consistency, construct validity, and content validity	*Assessment records* and *Interpretations*
Klein, 2013	DTZ	Bias and access	*Interpretations*
Cheng et al., 2020	CELPIP	Generalizability	*Interpretations*

### Assessment Records

Three studies (De Jong et al., 2009; Perlmann-Balme, 2011; Plassmann, 2011) provided evidence for *assessment records* inference. De Jong et al. (2009) developed an automated evaluation system of the TGN scoring. The TGN is a locally produced test assessing language learners’ Dutch proficiency. To probe the accuracy of the system, they compared machine-based scores and human ratings. Results showed a high correlation, indicating the machine scoring was reliable. Additionally, Perlmann-Balme (2011) and Plassmann (2011) investigated the DTZ, which is also a locally developed test used to examine test-takers’ German language proficiency. They found that the test experienced the processes of piloting, teacher feedback, and statistical analyses before it came out for actual use; and the real test papers were centrally rated through three steps (rater training, double rating, and post-test analyses) to ensure the score consistency.

It is found that there is a high correlation between machine scorings and human ratings of the TGN, and the DTZ has experienced several rounds of human ratings. These two backings support the sub-claim of score reliability in the AUA framework, and they both stress the significance of rater consistency.

### Interpretations

Five empirical studies (De Jong et al., 2009; Perlmann-Balme, 2011; Plassmann, 2011; Klein, 2013; Cheng et al., 2020) are linked to the *interpretations* inference. The current paper reviews the research in light of sub-dimensions in the AUA framework.

#### Meaningfulness

Perlmann-Balme (2011) and Plassmann (2011) investigated the construct and content validity of the DTZ. They found the DTZ developers worked out the construct based on the needs of test-takers by means of the Common European Framework of References for Languages levels (CEFR, Council of Europe, 2001), which is an international standard for measuring learners’ language ability widely adopted worldwide (Foley, 2021); and expert judgment was used to modify the construct and content of the test to ensure the test validity.

#### Impartiality

De Jong et al. (2009) examined the fairness of the TGN and concluded that test fraud was avoided in that tasks were randomly selected from a large benchmark. Also, test content and response formats were both fair to test-takers. Finally, bias was mitigated by piloting the test among different groups of people before actual use. However, a contradictory result was found in research of Klein (2013), which explored the fairness of the DTZ among different testing groups. It was concluded that some groups of test-takers (i.e., elderly people, Chinese, women with L1 Turkish, and men with L1 Russian and Polish) were more handicapped for taking the DTZ. It was suggested that more supports be given to test-takers during the test preparation stage.

#### Generalizability

Cheng et al. (2020) investigated the English language use situation among 14 participants who successfully immigrated to Canada in the workplace settings. All participants passed the CELPIP-General listening and speaking test. Some immigrants reported they had difficulty understanding different English accents. It was concluded that the communication strategies played an important role in mutual understandings; and new immigrants were positive about communicating and living in workplace settings.

#### Relevance and Sufficiency

In De Jong et al.’s (2009) research, it was reported that all information associated with immigration was public to immigration-seekers, which means they had the same access to test materials; and they could find the immigration requirements, especially language requirements, on the official website. However, immigration authorities failed to provide the justifications of the score cut-offs settings.

To apply research outcomes to the AUA framework, it could be sensed that the pertinent backings are as follows: the construct and content of the DTZ are based on needs analysis, and expert judgment has been employed for test modifications (meaningfulness); the TGN has fair test content, minimal test fraud and bias (impartiality), and enough test materials are offered to test-takers (relevance); and the CELPIP tasks well reflect the workplace situations (generalizability). However, potential rebuttals are also detected: the DTZ is relatively unfair among different testing groups due to their uneven language proficiency (impartiality); and reasons for the TGN cut-off score and corresponding decision are not given on the official website (sufficiency).

### Decisions

Only study of Hoang (2019) has touched upon the *decisions* inference, which investigated 39 test-takers taking IELTS or TOEFL for skilled migration purposes. Participants were invited to finish an online survey followed by an individual interview. Findings revealed test-takers’ perceptions varied from person to person. But more than half of them took a positive attitude towards test scores and believed the score was reliable; and they admitted the score-based decision was fair to them. This could be the backing to support the claim of decision equality in the AUA framework.

It might be right to claim that the use of large-scale proficiency tests such as IELTS and TOEFL is easier for policymakers to determine fair score-based decisions, because these kinds of tests have been extensively investigated and validated. Empirical research also suggests that most European countries, which adopt locally developed assessments, set the immigration language requirements largely based on CEFR levels varying from A1 to B2 levels (e.g., the German government asks immigration-seekers to obtain at least CEFR B1 in the DTZ; Van Avermaet, 2009). However, the utilization of CEFR may cause inequality problems because the CEFR scales are not specifically designed for assessing immigrants’ language skills, but broadly for evaluating English as a foreign language (EFL) learners’ language abilities (Krumm, 2007, North, 2009). Shohamy (2007, 2009) similarly argues that CEFR descriptors are not able to be used without modifications in that they could not be generalized to the immigration context.

In the current study, both DTZ and TGN offer CEFR levels on the score report for the government to set language requirements for immigration. However, no alignment research has been undertaken between scores of these locally developed tests and CEFR scales; and no official statistics are displayed in terms of applicants’ attitudes towards the fairness of decision-making by different countries.

### Consequences

Six empirical studies are associated with stakeholders’ perceptions of test consequences (Merrylees, 2003; Ahern, 2009; Merrifield, 2012; Rumsey et al., 2016; Frost, 2017; Hoang, 2019); and the stakeholders mainly comprise immigration-seekers, immigrants, and key personnel in the immigration authorities. Overall, stakeholders’ perceptions are categorized into *positive*, *negative*, and *compounded*.

#### Positivity

Merrylees (2003) probed the feasibility of the IELTS for immigration purposes from two groups of test-takers (who took the test for immigration and academic purposes). The method taken in the report was a survey questionnaire administered to 229 candidates. A major conclusion was that a positive impression was left on the test in terms of its appropriateness and effectiveness of the IELTS for immigration purposes; and test-takers from the immigration-seeking group generally believed the test was reliable. Besides, Merrifield (2012) explored the use of IELTS for immigration to Australia, New Zealand, Canada, and the United Kingdom from the immigration authorities’ perspective. A pure qualitative study was conducted through semi-structured interviews with key personnel in the immigration authorities. Results showed that immigration authorities generally accepted the decision-making; and New Zealand and Canada shared the most transparent and comprehensive decision-making systems.

#### Negativity

However, study of Rumsey et al. (2016) yields a contradictory result. They interviewed 14 health industry participants and 35 migrated health professionals in Australia. Health professionals all took IELTS for skilled migration purposes. Perceptions of the impact of IELTS on immigration purposes and practical use after the migration were covered in the interview. Results revealed that interviewees generally took a negative attitude towards IELTS as an immigration test. Some interviewees believed the scoring protocol was inconsistent and not relevant to their working environment and context. Additionally, Frost (2017) conducted an 18-month period study with in-depth and open-ended interviews among four individuals taking IELTS for skilled migration purposes in the Australian context about their perceptions in terms of the English test score requirements prescribed by the Australian government. Results indicated that “the individual subjectivities and the agency played an indispensable role in score meaning formulation and the consequences emerged over time from the use of test scores in Australia’s policy domain” (Frost, 2017, p. 246). The lack of validity and fairness of the use of English test scores for skilled migration purposes in Australia was perceived by migrant participants.

#### Compound

Ahern (2009) investigated participants’ perceptions of the IELTS test for its dual functions (education and immigration). A total of 12 students who attended IELTS preparation classes for immigration and higher education purposes at a local education agency were recruited in the study. The interview was used as a major research approach. The overall result was that students believed the impact of IELTS was compounded in that the stakes were quite high; and the consequences of the test doubled because the test may open the door for both higher education or residency, or block both situations. Furthermore, Hoang concluded that a few test-takers insisted that complex consequences, other than washback, may influence their test performance. Therefore, an assessment may not always carry positive or negative consequences to stakeholders, sometimes the consequences are complex.

Overall, the backings hinge on that immigration-seekers or immigrants believe the IELTS is reliable, and the decisions made by the government are relatively fair. But the rebuttals are illustrated that immigration-seekers or immigrants argue the scoring protocol of IELTS might be inconsistent; test items might be not quite relevant to real-life situations; and the validity and fairness of IELTS may still have room for improvement. To be noted, in the AUA framework, only beneficial and detrimental consequences that an assessment may bring about are stressed. But the importance of compounded consequences might be overlooked.

## Conclusion

The current paper reviewed 11 empirical studies related to validity issues of language tests for immigration purposes within AUA framework of Bachman and Palmer (2010). It was found that most research focused on stakeholders’ perceptions towards test consequences, i.e., the *consequences* inference. The stakeholders mainly include immigration-seekers or immigrants and key personnel in immigration authorities, which calls for further research to consider more kinds of stakeholders (e.g., test developers). Test developers are supposed to design authentic test papers. Whether items or contents are closely related to real-life situations or suitable for immigration purposes attach great indispensability to the validity of the test. Hence, the perceptions of test developers also merit academic research attention. Another innovative angle is to scratch the surface on the washback of an assessment brings about to the immigration-seekers. In other words, there is a need for further research to investigate whether immigration tests, especially locally produced tests, have effects on immigration-seekers’ foreign language learning. Moreover, more attention should be paid to immigration-seekers with uneven language proficiency about their attitudes towards the decision-making. Besides, only one study has touched upon immigration-seekers’ perceptions towards decisions, which advocates further research to examine the impartiality of decision-making, i.e., the *decisions* inference. Finally, the current AUA framework does not consider the compounded consequences an assessment may exert, which calls for further research to enrich the framework. It could be sensed that empirical studies related to language assessment for immigration are still not many in number. It is to be hoped further research could stress more on test validity issues.

In conclusion, this review paper sheds some light on the field of language assessment for immigration and brings about theoretical, practical, and political implications for various stakeholders. Theoretically, researchers or practitioners may better understand the topic of language assessment for immigration and the importance of validation research so that they could conduct more rigorous research. Besides, given the incompleteness of the AUA framework, they are encouraged to enrich the framework.

Practically, test developers may better know the drawbacks of either standardized or locally developed immigration tests so that they could design more authentic test items to ensure higher validity in test practices. Politically, policy or decision makers could better perceive immigration-seekers’ appeals so that they could make fairer immigration policies and decisions based on test scores.

## Author Contributions

DY and MW conceived the paper. DY took the lead in writing the manuscript. MW revised critically for important intellectual content and was in charge of the approval of the version to be published. All authors contributed to the article and approved the submitted version.

## Conflict of Interest

The authors declare that the research was conducted in the absence of any commercial or financial relationships that could be construed as a potential conflict of interest.

## Publisher’s Note

All claims expressed in this article are solely those of the authors and do not necessarily represent those of their affiliated organizations, or those of the publisher, the editors and the reviewers. Any product that may be evaluated in this article, or claim that may be made by its manufacturer, is not guaranteed or endorsed by the publisher.
